# Limited Impact of Short-Term Osteoporosis Medication on Vertebral Height Loss in the Acute Phase of Osteoporotic Vertebral Compression Fractures: A 3-Month Longitudinal Analysis

**DOI:** 10.3390/medicina62020299

**Published:** 2026-02-02

**Authors:** Jaehoon Kim, Bong-Ju Lee, Jae-Beom Bae, Sang-bum Kim, Dong-Hwan Kim, Ja-Yeong Yoon

**Affiliations:** 1 Department of Orthopaedic Surgery, Daejeon Sun Hospital, Daejeon 34811, Republic of Korea; superbdoc@hanmail.net (J.K.); bonjoolee@hanmail.net (B.-J.L.); bae.jaebeom@gmail.com (J.-B.B.); 2 Department of Orthopedic Surgery, Chungnam National University Sejong Hospital, Sejong 30099, Republic of Korea; sangbumos@me.com (S.-b.K.); kidhw411@gmail.com (D.-H.K.)

**Keywords:** osteoporotic fractures, spinal fractures, romosozumab, teriparatide, linear models, fractures, compression

## Abstract

*Background and Objectives*: The optimal pharmacological strategy to mitigate progressive vertebral collapse during the acute phase of osteoporotic vertebral compression fractures (OVCFs) remains a subject of debate. This initial 3-month window is the most critical period for evaluating the structural stability of the fracture, as the majority of progressive height loss occurs before solid bone union is achieved, directly influencing the decision to continue conservative management or transition to surgical intervention. *Materials and Methods*: In this retrospective study, 123 patients were allocated to control (*n* = 26), denosumab (*n* = 35), teriparatide (*n* = 30), or romosozumab (*n* = 32) groups. Treatment choice was non-randomized, driven by clinical pragmatism and patient preference. Serial changes in vertebral compression rate (VCR) and pain (VAS) were analyzed over 3 months using linear mixed models (LMMs) specifically adjusted for baseline imbalances in initial VCR. *Results*: In the unadjusted analysis, DMAB appeared to show a slower progression of compression compared to the control group. However, after adjusting for the initial VCR, no significant structural benefit was observed in any medication group (*p* > 0.05), with all groups showing small effect sizes (Cohen’s d < 0.4). In contrast, unstable fracture morphology was identified as the most potent driver of vertebral collapse (β = 2.758, 95% CI: 1.51–4.01, *p* < 0.001). Clinically, the RM group showed significantly lower overall pain levels throughout the follow-up period compared to the control group (*p* = 0.014). *Conclusions*: Short-term osteoporosis medication does not significantly mitigate vertebral collapse during the acute phase of OVCFs. Practically, these findings suggest that unstable fracture morphology and the baseline VCR—reflecting a potential ‘floor effect’ where less initially collapsed vertebrae may undergo more significant progression—are more informative predictors of acute collapse than medication choice. Consequently, early imaging-based risk stratification is crucial to identify patients at high risk for progressive deformity, regardless of their pharmacological regimen.

## 1. Introduction

With the rapidly aging population, osteoporotic vertebral compression fractures (OVCFs) have become a major public health burden. While the majority of these fractures heal with conservative management, a subset of patients experiences progressive vertebral collapse during the acute phase [1,2]. Such progressive collapse is clinically significant as it can lead to severe kyphotic deformity, persistent back pain, and potential neurological deficits due to bone fragment retropulsion [3]. Furthermore, failure of early mechanical stabilization may result in osteonecrosis, known as Kümmell’s disease, which often necessitates challenging surgical reconstruction [4]. Therefore, preventing excessive vertebral height loss in the early post-injury period is a primary goal of clinical management.

To prevent secondary fractures and promote healing, pharmacological intervention is essential. According to current guidelines [5], anabolic agents such as teriparatide (TPTD) and romosozumab (RM) are recommended for patients at high risk of fracture. Unlike antiresorptive agents, which primarily inhibit bone turnover, anabolic agents stimulate osteoblastic activity and have been shown to accelerate callus formation and bone union in clinical trials [6]. Theoretically, this rapid biological healing could provide early structural stability, provided that pharmacological management accounts for the distinct biological mechanisms and onset speeds of action for various agents [1,5,6]. For instance, romosozumab exerts a dual effect by rapidly increasing bone formation and decreasing resorption through sclerostin inhibition [5,6]. While teriparatide primarily stimulates osteoblastic activity to promote union [5,6], potent antiresorptives such as denosumab provide a rapid and sustained reduction in bone turnover [1,5]. Understanding these varying biological timelines is essential when evaluating acute-phase structural stability and preventing delayed complications like Kümmell’s disease [4] within the initial 3-month post-fracture window. Consequently, there is a growing clinical expectation that initiating potent anabolic therapy immediately after an OVCF diagnosis might prevent further deformity. However, a significant clinical gap exists regarding whether such early pharmacological intervention can meaningfully alter the rapid mechanical failure of a fractured vertebra. While potent agents offer biological plausibility by accelerating bone formation, and potentially enhancing bone strength, the mechanical reality—dominated by fracture instability—may dictate the degree of collapse before these pharmacological effects can fully manifest [7].

Therefore, it remains controversial whether these pharmacological benefits translate into the prevention of radiographic collapse during the acute phase (the initial 3 months). Most pivotal trials have focused on long-term bone mineral density (BMD) improvements or fracture risk reduction over 1 to 2 years, leaving a gap in evidence regarding short-term structural outcomes [8,9]. Moreover, in the acute setting, mechanical factors—such as the initial fracture morphology—may play a more dominant role than biological healing. For instance, Sugita et al. classified OVCFs based on initial imaging and suggested that specific “unstable” morphologies are predestined for poor prognosis regardless of standard treatment [10]. Despite this, few studies have directly compared the efficacy of various osteoporosis medications, including both anabolic and antiresorptive agents, against the natural course of vertebral collapse while simultaneously accounting for these baseline mechanical risk factors.

Therefore, the objective of this study was to investigate the factors influencing the progression of vertebral collapse during the initial 3-month acute phase of OVCF. We aimed to compare the radiographic protective effects of three distinct osteoporosis medications—TPTD, RM, and denosumab (DMAB)—against a control group. Furthermore, we sought to determine the relative contribution of pharmacological intervention versus baseline structural characteristics, specifically fracture morphology and initial compression severity, in determining the trajectory of vertebral height loss. Distinct from studies focused on long-term fracture prevention, this research specifically utilizes a 3-month observation window to evaluate whether pharmacological intervention can mitigate the precipitous mechanical collapse that typically occurs before significant biological bone remodeling can take place.

## 2. Materials and Methods

### 2.1. Patient Population

Ethical approval for this retrospective study was obtained from the Institutional Review Board (IRB) of Daejeon Sun Hospital (IRB No. [DSH-H-25-02]). This study was conducted in accordance with the ethical standards of the Declaration of Helsinki. The requirement for written informed consent was waived by the IRB due to the retrospective nature of the study.

This study reviewed 123 patients diagnosed with a single-level OVCF of the thoracolumbar spine (T10 or below) at Daejeon Sun Hospital between March 2022 and December 2023. Patients included in the study were postmenopausal women or men aged 50 years and older who were treatment-naïve, meaning they were first prescribed and treated with osteoporosis medication following their fracture diagnosis.

Patients were excluded if they had a follow-up period of less than one year. This inclusion criterion was established to ensure a consistent longitudinal dataset, facilitating a direct comparison between the primary acute-phase radiographic outcomes at three months and the secondary biological responses, BMD changes at one year. multi-level vertebral fractures (to prevent bias in compression rate measurement); fractures requiring surgical intervention (e.g., unstable fractures with three-column injury or neurological deficits); pathological fractures secondary to malignancy or infection; or secondary osteoporosis due to conditions such as diabetes mellitus, long-term steroid use, or chronic renal failure. Furthermore, patients who used anabolic agents for longer than three months were excluded, as this patient subgroup was too small for meaningful analysis and could introduce bias.

### 2.2. Diagnosis and Assessment

The diagnosis of an acute OVCF was confirmed in all patients via magnetic resonance imaging (MRI). The diagnosis was established by identifying characteristic signal changes within the fractured vertebral body, specifically low signal intensity on T1-weighted images and corresponding high signal intensity on T2-weighted or fat-suppressed T2-weighted images. These findings were indicative of bone marrow edema consistent with a recent fracture [11].

Baseline BMD was measured with a dual-energy X-ray absorptiometry (DEXA) scan (Horizon, Hologic Inc., Bedford, MA, USA). A follow-up DEXA scan was performed one year after the initial diagnosis to evaluate changes in BMD.

### 2.3. Treatment Protocol

Following diagnostic confirmation, all patients were treated conservatively and fitted with a thoracolumbar orthosis (TLSO), which they were instructed to wear for at least three months [2]. Additionally, all patients, including those in the control group, received standardized instructions for calcium and vitamin D supplementation (e.g., 500 mg of calcium and 1000 IU of vitamin D daily) as part of routine osteoporosis care. For pharmacological intervention, anabolic agents were recommended as the first-line therapy to all patients, consistent with the Endocrine Society Clinical Practice Guidelines for patients at high to very-high risk of fracture [12]. Teriparatide (TPTD; Forsteo; Eli Lilly and Company, Indianapolis, IN, USA) was the primary choice due to its potential to aid physiological fracture healing [13]. If patients declined the daily injections required for TPTD, monthly romosozumab (RM; Evenity; Amgen Inc., Thousand Oaks, CA, USA) was offered as an alternative to maintain the therapeutic strategy of using potent anabolic agents. Patients who refused all offered pharmacological treatment options from baseline were assigned to the control group, representing true untreated controls. A bisphosphonate (BP) group was not included in this study because, while zoledronic acid had previously been a primary treatment option at our institution, its clinical use has become negligible following the widespread clinical adoption of denosumab (DMAB; Prolia; Amgen Inc., Thousand Oaks, CA, USA). To minimize misclassification bias, only patients in the medication groups who maintained their prescribed regimen for at least the initial 3-month observation window—verified through electronic prescription records—were included in the final analysis. Those who discontinued therapy within the first 3 months due to adverse events or other constraints were excluded.

#### Treatment Allocation

Treatment assignment was non-randomized and was primarily determined by a combination of clinical pragmatism and patient-centered factors. While physician recommendations initially considered fracture severity and risk of collapse, the final treatment allocation was predominantly driven by socioeconomic considerations and patient preferences rather than purely by clinical indications. Specifically, the financial burden associated with domestic health insurance reimbursement limitations, tolerance for injection frequency (daily vs. monthly), and overall cost-effectiveness were the decisive factors in the final selection of the pharmacological agent. Consequently, the distribution of patients across medication groups reflects these pragmatic real-world constraints, which were accounted for in our subsequent statistical adjustments to ensure a valid comparison of effectiveness

### 2.4. Radiologic Assessment

#### 2.4.1. Follow-Up and Compression Rate Measurement

During the standard two-week inpatient treatment period, serial plain radiographs of the spine were obtained at baseline, one week, and two weeks post-injury. Subsequent follow-up radiographs were taken at one, two, and three months on an outpatient basis.

All radiographs were consistently obtained in the supine position throughout the study period. This approach was primarily adopted to ensure patient safety and minimize the risk of aggravating structural instability during the acute phase of the fracture, where severe pain often precludes standing imaging. Furthermore, using a standardized supine position ensured the comparability of radiographic measurements across all follow-up intervals. To ensure objectivity, the radiographic assessments were performed by an independent observer who was blinded to the treatment group allocation and clinical information.

**Figure 1:**
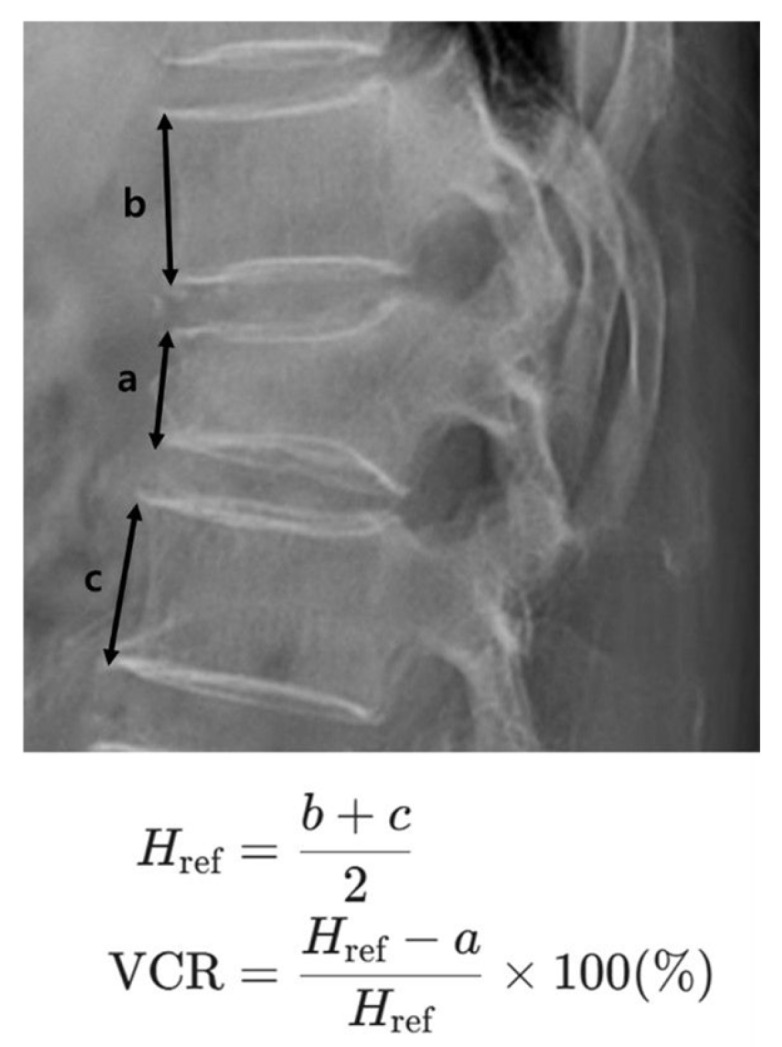
Measurement of the Vertebral Compression Rate (VCR). The anterior vertebral height of the fractured body is measured as a. The anterior heights of the adjacent superior and inferior vertebral bodies are measured as b and c, respectively. The reference height (Href) is calculated as the average of the adjacent vertebral heights [Href = (b + c)/2]. The VCR is then calculated using the formula: VCR (%) = (1 − a/Href) × 100, representing the percentage of height loss relative to the estimated normal height.

On each radiograph, the anterior height (AH) of the fractured vertebral body was measured. To calculate the vertebral compression rate (VCR), this value was compared to a reference height. This reference height was determined by averaging the anterior heights of the vertebrae directly above and below the fractured one. The VCR was then calculated as the percentage loss of height of the fractured vertebra relative to this reference height. Thus, a higher VCR value indicates a more severe loss of anterior vertebral height (Figure 1).

#### 2.4.2. Fracture Type Classification

Based on initial plain radiographs and computed tomography (CT) scans, all fractures were classified into five morphological types according to the system described by Sugita et al. [10]: Swelled, Bow-shaped, Projecting, Concave, and Dented.

**Figure 2:**
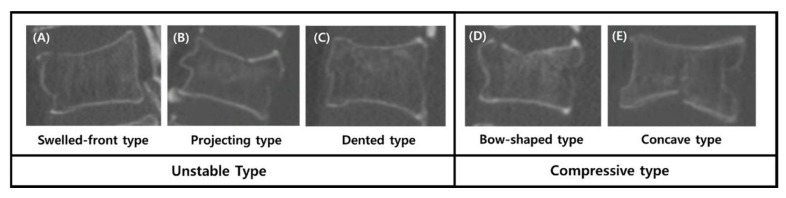
Morphological classification of osteoporotic vertebral compression fractures based on computed tomography (CT) scans. Representative mid-sagittal CT images illustrating the five fracture types described by Sugita et al. [10] (**A**) Swelled-front type, exhibiting swelling of more than 50% of the anterior vertebral wall. (**B**) Projecting type, showing a distinct anterior cortical fragment protruding forward. (**C**) Dented type, characterized by a focal fracture line or dent in the center of the anterior cortex, (**D**) Bow-shaped type, demonstrating a broad depression of the endplate resembling a ship’s bow, with the anterior wall pinched in. (**E**) Concave type, showing a focal depression confined to the center of the endplate. Based on the prognostic implications for instability in this study, these five types were re-categorized into two groups: the ‘Unstable type’ (**A**–**C**), which includes fractures with anterior cortical bulging, projection, or disruption, and the ‘Compressive type’ (**D**,**E**), which represents fractures characterized primarily by vertical endplate depression without anterior protrusion. All radiographic measurements and fracture classifications were performed by a single independent observer (an orthopedic surgeon). To ensure the reliability of these assessments, intra-observer reliability was evaluated by re-measuring 20 randomly selected cases four weeks after the initial assessment. The intraclass correlation coefficient (ICC) for VCR measurements was 0.96, and the weighted kappa for Sugita classification was 0.95, both indicating excellent consistency. Furthermore, to satisfy the requirement for objective validation, any ambiguous cases were reviewed and finalized through consensus with a senior spine surgeon, achieving an inter-observer agreement level equivalent to an ICC of 0.95.

However, as some fracture types had very few patients (e.g., Swelled, *n* = 9; Dented, *n* = 8), the fractures were re-categorized into two broader groups to facilitate parametric statistical analysis. This re-categorization was based on shared morphological features and their associated prognostic implications for instability. The Swelled, Projecting, and Dented types, which are characterized by significant disruption or bulging of the anterior vertebral cortex, were grouped as the ‘unstable type’ as they are associated with a higher risk of progressive collapse. Conversely, the Bow-shaped and Concave types, which represent fractures with a more established compression pattern often involving an intact or already compacted anterior cortex, were grouped as the ‘compressive type’ (Figure 2).

### 2.5. Clinical Outcome and Complication Assessment

Clinical outcomes were assessed using the Visual Analog Scale (VAS) for back pain. VAS scores were recorded at the initial visit, and at 1-month and 3-month follow-up visits. The reduction in VAS score from baseline to 3 months was calculated to evaluate the efficacy of pain relief.

### 2.6. Assessment of Intravertebral Vacuum Cleft (IVC)

In this study, the appearance of a new intravertebral vacuum cleft (IVC) on plain radiographs at the 3-month follow-up was utilized as a radiographic indicator of progressive instability and potential osteonecrosis. While a definitive diagnosis of delayed vertebral osteonecrosis typically requires a more prolonged clinical course, the presence of an IVC is a well-established hallmark of progressive vertebral collapse [14,15]. This approach allowed us to objectively describe the clinical trajectory of patients demonstrating progressive collapse during the acute phase.

### 2.7. Statistical Analysis

**Figure 3:**
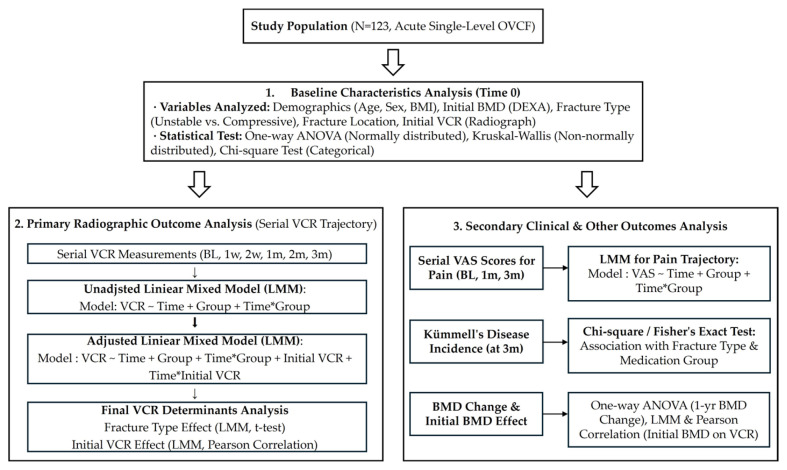
Flowchart of the statistical analysis process. The study followed a systematic analytical approach divided into baseline assessment, primary radiographic outcome analysis, and secondary clinical outcome analysis. Initially, baseline variables including demographics, bone mineral density (BMD), and fracture characteristics were compared across the four treatment groups at Time 0 (baseline) using one-way ANOVA, Kruskal–Wallis, or Chi-square tests to verify homogeneity. The primary outcome analysis evaluated the trajectory of the vertebral compression rate (VCR) measured at baseline (BL), 1 week (1 w), 2 weeks (2 w), and 1, 2, and 3 months (1 m, 2 m, 3 m). This involved an unadjusted Linear Mixed Model (LMM) followed by an adjusted LMM that controlled for initial VCR severity to identify the final determinants of vertebral collapse, such as fracture morphology and initial compression degree. Finally, secondary clinical and other outcomes were assessed, including trajectories of pain relief via Visual Analog Scale (VAS) scores at BL, 1 m, and 3 m using LMM, as well as the 3-month incidence of an IVC through Chi-square or Fisher’s exact tests. One-year BMD changes and the potential influence of initial BMD on VCR progression were also analyzed using one-way ANOVA, LMM, and Pearson correlation. Abbreviations: BL, baseline; w, week(s); m, month(s); OVCF, osteoporotic vertebral compression fracture; BMD, bone mineral density; VCR, vertebral compression rate; LMM, linear mixed model; VAS, visual analog scale; DEXA, dual-energy X-ray absorptiometry.

The overall schematic diagram of the study design and analytical workflow is presented in Figure 3. All statistical analyses were performed using R software (version 4.2.0; R Foundation for Statistical Computing, Vienna, Austria).

#### 2.7.1. Analysis of Baseline Characteristics

Baseline demographic and clinical characteristics were compared among the different treatment groups. Categorical variables, such as sex and fracture location, were analyzed using the chi-square test. For continuous variables like age, height, and weight, normality was assessed using the Shapiro–Wilk test. Normally distributed data were compared using an independent *t*-test or one-way analysis of variance (ANOVA), while non-normally distributed data were compared using the Mann–Whitney U test or Kruskal–Wallis test.

#### 2.7.2. Analysis of the Primary Outcome (Medication Effects)

To assess the effect of medication on VCR, the analysis was conducted in three steps. First, the overall change in VCR from baseline to 3 months was compared among the four treatment groups using a one-way ANOVA.

Second, to analyze the trajectory of VCR progression over the entire 3-month follow-up period, an unadjusted linear mixed model (LMM) was used, where time, medication group, and the time-by-medication interaction were included as fixed effects. Third, an adjusted LMM was performed to control the confounding effect of baseline severity. Given the significant group differences in initial VCR, the initial VCR and its interaction with time (Time X Initial VCR) were included as covariates to evaluate the independent effect of medication. The LMM was specified with a random-intercept structure to account for the inherent correlation between repeated measurements within the same individual. Time was modeled as a categorical fixed factor (baseline, 1, 2, and 3 months) to capture potential non-linear trends in vertebral collapse. To handle missing data, we utilized the Full Information Maximum Likelihood (FIML) estimation method, which provides unbiased parameter estimates under the assumption that data are missing at random (MAR), thereby preserving the original sample size without the need for simple imputation. To quantify the clinical significance of the observed differences, Cohen’s d effect sizes were calculated for each medication group relative to the control group.

Furthermore, a change of >10% in the VCR was defined as the minimal clinically important difference (MCID), based on its established association with progressive kyphotic deformity. Finally, the change in spine BMD T-score over one year was assessed by calculating the difference between the baseline and one-year follow-up values, followed by a one-way ANOVA to compare mean changes among groups.

#### 2.7.3. Analysis of Prognostic Factors on VCR Progression

To identify baseline factors associated with VCR progression, several secondary analyses were performed. The effect of fracture type (Unstable vs. Compressive) was evaluated using an independent *t*-test and an LMM. Furthermore, the effect of the initial VCR on the subsequent change in VCR over time (ΔVCR) was evaluated using both Pearson correlation analysis and an LMM. Finally, the effect of initial BMD on VCR change was also analyzed using Pearson correlation and an LMM.

#### 2.7.4. Analysis of Clinical Outcomes and Complications

Changes in VAS scores over time were analyzed using LMM with time, medication group, and their interaction as fixed effects. The Control group was set as the reference to evaluate the relative efficacy of each medication. The mean reduction in VAS scores among groups was compared using one-way ANOVA. Additionally, the correlation between initial VCR and VAS reduction was analyzed using the Pearson correlation test.

The incidence of IVC formation disease was compared among medication groups and fracture types using the Chi-square test or Fisher’s exact test, as appropriate. Independent *t*-tests were used to compare the initial VCR between patients who developed IVC formation and those who did not.

#### 2.7.5. Power Analysis

A post hoc power analysis was performed to evaluate the statistical power of the study using G*Power software (version 3.1.9.7). For a one-way ANOVA with four groups (*n* = 123) and a medium-to-large effect size (Cohen’s f = 0.30), the calculated power was 0.82 at a significance level of α = 0.05. This indicates that our sample size was sufficient to detect clinically meaningful differences in vertebral compression rate (VCR) among the treatment groups.

## 3. Results

### 3.1. Patient Demographics and Baseline Characteristics

A total of 123 patients were included in the final analysis and were stratified into four treatment groups: the control group (*n* = 26), the DMAB group (*n* = 35), the TPTD group (*n* = 30), and the RM group (*n* = 32).

There were no statistically significant differences in baseline demographic data—including age, sex, height, weight, initial spine BMD, fracture group, and fracture location—among the four medication groups (*p* > 0.05 for all comparisons) (Table 1). These findings confirm that the study groups were homogeneous, allowing for a comparable analysis.

**Table 1:** Baseline demographics and clinical characteristics by medication group.

Variable	RM (*n* = 32)	TPTD (*n* = 30)	DMAB (*n* = 35)	Control (*n* = 26)	*p*-Value	SMD ***
Age (years)	73.19 ± 7.35	75.63 ± 8.76	78.17 ± 8.39	74.38 ± 10.79	0.1231	0.55
Height (cm)	157.45 ± 6.86	156.00 ± 5.36	155.20 ± 7.22	159.00 ± 8.16	0.0627	0.51
Weight (kg)	59.10 ± 9.48	60.07 ± 7.78	56.30 ± 9.65	58.83 ± 9.48	0.4135	0.24
Initial BMD (Spine T-score)	−2.46 ± 1.04	−2.39 ± 1.02	−2.85 ± 0.93	−2.63 ± 0.98	0.2097	0.46
Sex (*N*, %)					0.2251	0.31
Male	5 (15.6%)	1 (3.3%)	3 (8.6%)	5 (19.2%)		
Female	27 (84.4%)	29 (96.7%)	32 (91.4%)	21 (80.8%)		
Fracture Group (*N*, %) *					0.2667	0.41
Compressive	26 (81.2%)	18 (60.0%)	26 (74.3%)	17 (65.4%)		
Unstable	6 (18.8%)	12 (40.0%)	9 (25.7%)	9 (34.6%)		
Fracture Location (Level) **	N/A	N/A	N/A	N/A	0.3739	
Initial VCR (%) †	14.73 ± 10.21	15.52 ± 9.84	25.14 ± 14.28	21.35 ± 13.56	0.003	0.84

Data are presented as mean ± standard deviation (SD) for continuous variables or as count (*N*, %) for categorical variables. *p*-values were calculated using the Kruskal–Wallis test for continuous variables and the Chi-Square test for categorical variables. Statistical significance was set at *p* < 0.05. Abbreviations: RM, Romosozumab; TPTD, Teriparatide; DMAB, Denosumab; BMD, Bone Mineral Density; N/A, Not Applicable. * Fracture Group was re-categorized based on the Sugita et al. classification (FractureType1 column): ‘Compressive’ (Types 2, 4) and ‘Unstable’ (Types 1, 3, 5). ** Fracture Location (Level) includes 11 distinct anatomical levels (T10-L5). Detailed group breakdowns (*N*, %) are omitted for clarity; the *p*-value represents the overall comparison of distribution across groups. *** SMD (Standardized Mean Difference) values > 0.1 indicate potential baseline imbalance. The large SMD for Initial VCR (0.84) justifies the use of adjusted statistical models. † Initial VCR differed significantly among groups (*p* = 0.003) and was incorporated as a primary covariate in the adjusted Linear Mixed Model (Table 2). The higher initial VCR observed in the denosumab group reflects a potential selection bias in real-world clinical practice, where antiresorptive agents may have been preferred for vertebrae with more severe initial collapse.

**Table 2:** Linear mixed model analysis of VCR progression: unadjusted versus adjusted models.

Predictor	Unadjusted Model (*p*-Value) ^a^	Adjusted Model (β [95% CI]) ^b^	*p*-Value
Time (Natural Progression)	<0.001 *		<0.001 *
Medication Interaction ^c^			
Time × DMAB	0.010 *	−0.339 [−1.43, 0.75]	0.544
Time × RM	0.752	0.132 [−1.41, 1.67]	0.868
Time × TPTD	0.086	−0.672 [−2.24, 0.90]	0.406
Covariates			
Time × Initial VCR ^d^	-	−0.033 [−0.05, −0.01]	0.003 *

Abbreviations: β, beta coefficient (estimated effect size); CI, confidence interval; VCR, Vertebral Compression Rate; DMAB, Denosumab; RM, Romosozumab; TPTD, Teriparatide. * Indicates statistical significance (*p* < 0.05). ^a^ Unadjusted Model: This model analyzes the effect of medication on VCR progression with time, without considering baseline differences in fracture severity. ^b^ Adjusted Model: This model includes the ‘Initial VCR’ as a covariate to control for the baseline imbalance in fracture severity among groups. ^c^ Medication Interaction (Time × Drug): This term represents the difference in the rate of progression (slope) between a specific medication group and the Control group. A significant *p*-value indicates that the medication significantly altered the speed of vertebral collapse compared to the natural course (Control). ^d^ Time × Initial VCR: This interaction term evaluates whether the baseline severity (Initial VCR) influences the rate of future progression. A significant *p*-value (0.003) indicates that the initial severity, rather than the medication type, was the primary factor determining the trajectory of collapse (i.e., a ‘floor effect’ where severe fractures progress more slowly).

### 3.2. Effect of Medication on Vertebral Compression Rate and Bone Mineral Density

Analysis of the overall VCR change from baseline to 3 months revealed no statistically significant difference among the four groups (One-way ANOVA, *p* = 0.3898). The mean (±SD) change was 24.90 ± 17.46 for the RM group, 21.87 ± 16.34 for the TPTD group, 19.81 ± 18.86 for the DMAB group, and 27.08 ± 17.26 for the control group.

In the unadjusted LMM analysis, a statistically significant time-by-medication interaction effect was initially observed for the DMAB group compared to the Control group (β = −1.331; 95% CI: −2.34 to −0.32; *p* = 0.010), suggesting a slower progression of compression in the DMAB group.

As shown in Table 1, the DMAB group had a significantly higher initial VCR (25.14 ± 14.28%) compared to other groups (*p* = 0.003), indicating a potential selection bias. To account for this baseline imbalance, an adjusted LMM analysis was performed by treating the initial VCR as a covariate. After adjusting for this factor, the previously observed protective effect of DMAB was no longer statistically significant (β = −0.339; 95% CI: −1.43 to 0.75; *p* = 0.544), and no medication group showed a significant structural benefit compared to the Control group. Instead, the interaction between time and initial VCR was highly significant (β = −0.033; 95% CI: −0.05 to −0.01; *p* = 0.003), confirming that the initial severity of compression, rather than the medication type, was the primary driver of the VCR trajectory.

In the analysis of bone density, the 1-year change in spine BMD T-score also showed no statistically significant difference among the groups (One-way ANOVA, *p* = 0.5478). The mean (±SD) T-score change was 0.56 ± 0.62 for the RM group, 0.58 ± 0.44 for the TPTD group, 0.50 ± 0.54 for the DMAB group, and 0.14 ± 0.59 for the control group.

In summary, while the unadjusted analysis indicated a significant difference in VCR progression for the DMAB group, this significance was no longer observed after adjusting for the initial VCR. In the final adjusted model, only the interaction between time and initial VCR remained statistically significant (*p* = 0.003), whereas the interaction between time and medication did not. Similarly, no significant difference was observed in the 1-year change in spine BMD among the medication groups.

The calculated Cohen’s d effect sizes for the 3-month VCR change compared to the control group were 0.12 for the RM group, 0.31 for the TPTD group, and 0.40 for the DMAB group. Despite these trends, none of the medication groups reached the defined MCID threshold of 10% after covariate adjustment, further supporting the limited structural impact of pharmacological intervention during the acute phase.

In summary, our adjusted LMM identifies a significant ‘floor effect’ in vertebral progression (β = −0.033, 95% CI: −0.05 to −0.01, *p* = 0.003). This concept suggests that vertebrae with a higher initial compression rate have a naturally lower potential for additional height loss, as the trabecular bone is already significantly compacted. Conversely, fractures with low initial VCR are at the highest risk for precipitous collapse, emphasizing that the initial degree of severity—rather than the pharmacological regimen—is the primary determinant of the 3-month structural trajectory.

### 3.3. Effect of Fracture Type on Vertebral Compression Rate

To evaluate the impact of fracture morphology on VCR progression independently of medication, the cohort was stratified into the Unstable type (*n* = 36) and the Compressive type (*n* = 87) based on the Sugita classification.

An independent *t*-test revealed that the Unstable group experienced a significantly greater increase in the VCR during the first two weeks of follow-up. The mean increase in VCR for the Unstable group was significantly higher during both the interval between baseline and the one-week follow-up (12.06 ± 14.55 vs. 4.83 ± 7.46; *p* = 0.0004) and the interval between the one-week and two-week follow-ups (7.19 ± 6.30 vs. 2.99 ± 5.83; *p* = 0.0006). Notably, the Unstable group experienced an additional cumulative height loss of 11.43% compared to the Compressive group within the first 14 days, representing a substantial clinical disparity in acute structural failure.

**Figure 4:**
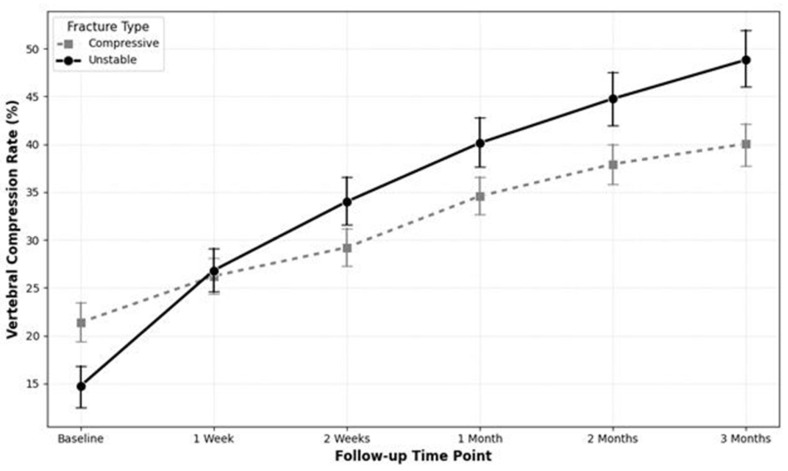
Trajectory of vertebral compression rate (VCR) progression over time according to fracture morphology (Sugita classification). This graph compares the progression of absolute VCR (%) between the Unstable fracture type (solid line with circle markers) and the Compressive fracture type (dashed line with square markers) over the 3-month follow-up period. The Unstable group demonstrates a significantly steeper increase in VCR, particularly during the acute phase (from baseline to 2 weeks), compared to the Compressive group. This visual pattern corresponds with the statistical finding that unstable fracture morphology is a critical prognostic factor for rapid vertebral collapse. Error bars represent the standard error of the mean (SEM).

An LMM analysis assessing the entire 3-month trajectory confirmed this finding. A significant time-by-group interaction effect was observed (β = 2.758; 95% CI: 1.51 to 4.01; *p* < 0.0001), indicating that the Unstable group had a statistically significant and more rapid progression of vertebral compression over time compared to the Compressive group (Figure 4).

### 3.4. Relationship Between Initial and Subsequent Changes in Compression Rate

**Figure 5:**
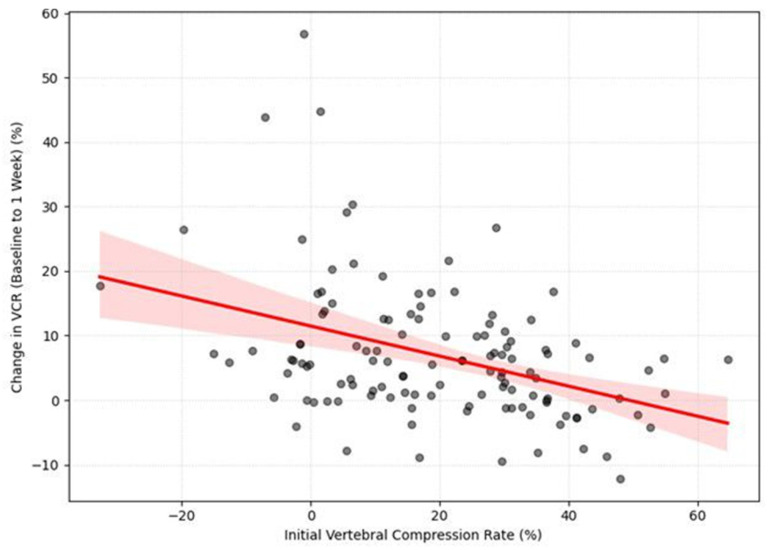
Scatter plot illustrating the negative correlation between initial vertebral compression rate (VCR) and the subsequent progression during the first week. The scatter plot demonstrates a statistically significant negative correlation (r = −0.395, *p* < 0.001) between the initial VCR (*x*-axis) and the change in VCR from baseline to 1 week (*y*-axis). This indicates that patients with a higher initial compression rate experienced a smaller magnitude of additional collapse during the acute phase, suggesting a potential ‘floor effect’ in vertebral progression. The red line represents the linear regression fit, illustrating the significant negative correlation where a higher initial compression severity is associated with a smaller magnitude of subsequent height loss.

Next, we examined the relationship between the initial VCR and its subsequent progression. A Pearson correlation analysis revealed a significant negative correlation between the initial vertebral compression rate (VCR) and the subsequent change in VCR (ΔVCR) during the first week of follow-up (r = −0.395, *p* < 0.001). (Figure 5) This indicates that a higher initial compression rate was associated with a smaller amount of subsequent compression change. The negative correlation was still significant from week 1 to week 2, although it was weaker (r = −0.189, *p* = 0.037). After two weeks, no significant correlations were found (*p* > 0.05).

The LMM analysis further clarified these temporal dynamics. There was a significant negative effect of time on the ΔVCR (β = −1.911, *p* < 0.001), indicating that the vertebra compressed the most during the first few weeks, and then the rate of further compression slowed down. Furthermore, the LMM revealed a significant negative main effect of the initial VCR on the overall change in compression (β = −0.178, *p* < 0.001).

Finally, the LMM revealed a significant interaction effect between time and the initial VCR (β = 0.052, *p* < 0.001). This finding indicates that the rate of compression change over time was different for patients with high versus low initial compression rates. Specifically, patients with a low initial VCR experienced a rapid progression of compression in the early stages, which then decelerated over time. In contrast, patients who presented with a high initial VCR from the outset showed a more gradual and steady pattern of compression change.

### 3.5. Relationship Between Initial Bone Mineral Density and Change in Compression Rate

The influence of initial bone mineral density (BMD) on VCR change was also assessed. A Pearson correlation analysis was conducted to assess the direct linear relationship between initial spine bone mineral density (BMD) and the subsequent change in the vertebral compression rate (VCR). No significant correlation was observed between these two variables. Specifically, the correlation between initial BMD and the change in VCR from baseline to 1 week (r = −0.129, *p* = 0.155) and from 1 week to 2 weeks (r = 0.038, *p* = 0.678) were both non-significant.

To further evaluate the influence of initial BMD on the VCR over the entire follow-up period, an LMM analysis was performed. The results of the LMM analysis showed that there was no significant main effect of initial BMD on the change in VCR (β = −0.551, *p* = 0.316). Furthermore, the interaction effect between initial BMD and time was also not statistically significant (β = 0.238, *p* = 0.279).

### 3.6. Clinical Outcomes: VAS Scores for Back Pain

All treatment groups demonstrated significant pain relief over the 3-month follow-up period (ANOVA, *p* = 0.002). In terms of the absolute reduction in VAS scores from baseline to 3 months, the TPTD group showed the largest mean reduction (6.87 ± 0.78), followed closely by the RM group (6.66 ± 0.97).

**Figure 6:**
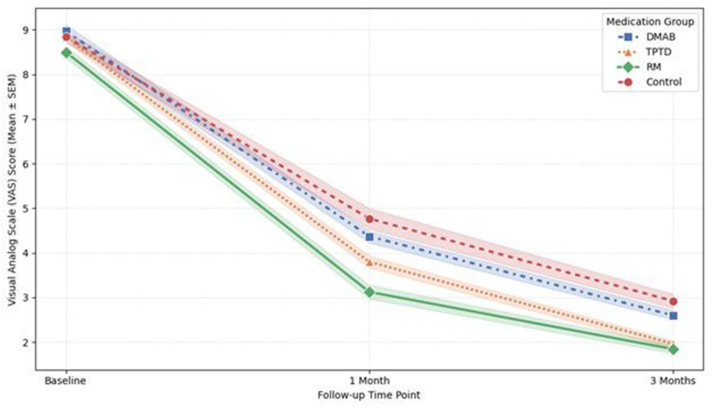
Trajectories of Visual Analog Scale (VAS) scores for back pain over the 3-month follow-up period by medication group.

However, the LMM analysis, which evaluates the overall pain trajectory throughout the treatment period, revealed that the RM group was the only group to show a statistically significant difference compared to the Control group (Coef. = −0.797, *p* = 0.014). This indicates that patients treated with RM experienced significantly lower overall pain levels during the follow-up period compared to those in the Control group. In contrast, neither the TPTD (*p* = 0.137) nor the DMAB (*p* = 0.482) group showed a statistically significant difference in pain trajectory compared to the Control group (Figure 6).

Additionally, a positive correlation was observed between the initial VCR and the reduction in VAS score (r = 0.307, *p* < 0.001), indicating that patients with more severe initial compression experienced a greater magnitude of pain relief. No significant difference in pain reduction was found based on fracture morphology (*p* = 0.434).

The line graph illustrates the changes in mean VAS scores at baseline, 1 month, and 3 months for each treatment group. While all groups exhibited a reduction in pain over time, the linear mixed model (LMM) analysis revealed that the Romosozumab (RM) group (solid line with diamond markers) maintained significantly lower overall pain levels throughout the follow-up period compared to the Control group (dashed line with circle markers) (*p* = 0.014). In contrast, the Denosumab (DMAB) and Teriparatide (TPTD) groups did not show a statistically significant difference in pain trajectory compared to the Control group. Error bars represent the standard error of the mean (SEM).

### 3.7. Incidence of IVC Formation

**Figure 7:**
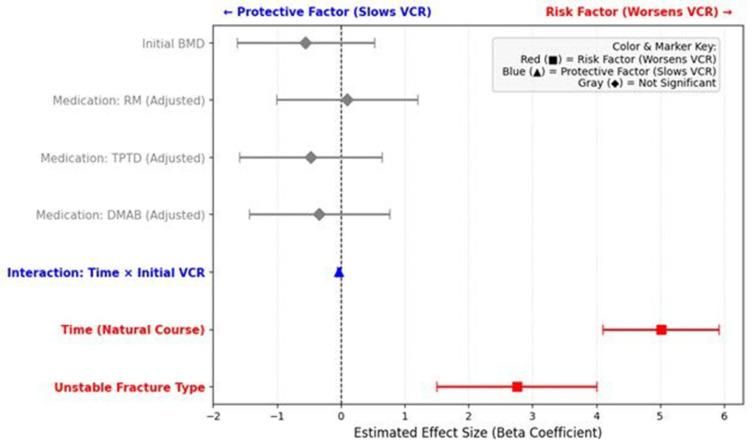
Forest plot summarizing the estimated effect sizes (Beta coefficients) of prognostic factors and medication on the progression of vertebral compression rate (VCR). The plot displays the Beta coefficients and 95% confidence intervals (CI) derived from the linear mixed model (LMM) analysis. The Red Squares (■) indicate significant risk factors (positive Beta) that accelerate vertebral collapse. Specifically, the ‘Unstable fracture type’ (compared to the Compressive type) and the natural course of ‘Time’ were identified as major contributors to rapid VCR progression. The Blue Triangle (▲) represents a significant protective factor (negative Beta) that mitigates progression. This corresponds to the interaction between ‘Time’ and ‘Initial VCR’, indicating a ‘floor effect’ where higher initial compression slows down further collapse. The gray diamonds (◆) indicate factors that were not statistically significant (95% CI crossing zero). Notably, after adjusting for baseline severity, none of the medication groups (DMAB, RM, TPTD) showed a significant effect on VCR progression compared to the Control group.

At the 3-month follow-up, radiological evidence of potential osteonecrosis, indicated by the presence of an IVC, was identified in 8 out of 123 patients (6.5%). The incidence of IVC formation did not differ significantly among the medication groups (*p* = 0.489). However, a significant association was found between fracture morphology and the development of an IVC. Patients with Unstable type fractures had a significantly higher incidence of IVC formation compared to those with Compressive type fractures (5/36 [13.9%] vs. 3/87 [3.4%], Fisher’s exact test, *p* = 0.047). No significant difference was observed in the initial VCR between patients who developed an IVC and those who did not (16.14% vs. 19.68%, *p* = 0.591). To summarize the overall impact of the investigated factors on vertebral compression progression, the estimated effect sizes (Beta coefficients) for medication, fracture type, and initial severity are visually synthesized in a forest plot (Figure 7).

## 4. Discussion

This study investigated the factors influencing the progression of vertebral compression during the critical first three months following an OVCF [16]. Specifically, we first evaluated the impact of different pharmacological interventions—including anabolic agents and antiresorptives—and subsequently analyzed the influence of baseline prognostic factors such as fracture morphology and initial severity. Our analysis demonstrated that the type of anti-osteoporotic medication did not significantly mitigate the radiographic progression of vertebral collapse during this acute period. Instead, the morphological type of the fracture and the initial severity of compression were identified as the primary determinants influencing further vertebral compression.

Several previous studies have suggested that anabolic agents, such as TPTD and RM, can accelerate fracture healing and prevent progressive collapse in OVCFs. [17,18] For instance, a randomized controlled trial by Aspenberg et al. demonstrated that TPTD improved radiographic fracture healing compared to placebo [19]. Similarly, RM has been shown to rapidly increase bone mineral density and reduce fracture risk [20]. Based on these findings, it was hypothesized that early administration of anabolic agents would mitigate vertebral height loss in the acute phase. However, contrary to these expectations, our study found that the type of medication did not significantly alter the structural trajectory of vertebral collapse over the 3-month follow-up. This discrepancy may be attributed to the duration of treatment. Most studies demonstrating the structural benefits of anabolic agents involved treatment periods of at least 6 to 12 months [21]. In the acute phase (first 3 months), the biological process of callus formation and mineralization may not be sufficient to counteract the mechanical forces causing collapse, regardless of the potent anabolic stimulus. This suggests that while anabolic agents are effective for long-term bone quality, their ability to mechanically stabilize an acute fracture within a short timeframe may be limited.

While pivotal long-term studies, such as the ARCH and FRAME trials, have established the superior efficacy of anabolic agents in increasing bone mineral density (BMD) and reducing fracture risk over 12 to 24 months, our findings highlight a critical temporal disconnect in the acute phase [9,20]. The biological onset of increased bone formation and subsequent mineralization typically requires several months to translate into enhanced structural stiffness [21,22]. In contrast, the mechanical failure of a fractured vertebra occurs most precipitously within the first few weeks post-injury, driven by immediate weight-bearing forces [10,16]. Therefore, even the most potent anabolic stimulus may be insufficient to counteract this early ‘mechanical window’ of instability before significant callus formation can manifest [22].

A pivotal finding of this study is that the apparent protective effect of medication observed in the initial analysis was attributable to baseline variances rather than pharmacological efficacy. To overcome the limitations of simple cross-sectional comparisons and accurately track the longitudinal trajectory of vertebral collapse, we employed an LMM analysis. In our unadjusted LMM, DMAB initially appeared to slow the progression of vertebral collapse. However, after adjusting for the initial vertebral compression rate (VCR) within the LMM framework, this protective effect disappeared. The DMAB group had a significantly higher initial VCR, suggesting that the observed stability was likely due to a ‘floor effect’—where severe fractures have less trabecular bone remaining to collapse further—rather than a direct benefit of the drug. This finding underscores the limitations of short-term (3-month) pharmacological intervention in altering the structural course of acute fractures. Several studies have demonstrated that anabolic agents, such as TPTD and RM, require a longer duration (typically 6 to 12 months) to stimulate sufficient callus formation and mineralization to counteract mechanical loading [22]. Therefore, in the acute phase, the biological process of bone formation may not be rapid enough to prevent mechanical collapse caused by weight-bearing forces.

The observed clinical dissociation between pain relief and the absence of structural protection in the RM group may suggest a need for further mechanistic exploration. One possible explanation is that RM’s unique dual action—simultaneously promoting bone formation and inhibiting resorption might influence local bone turnover dynamics. It is conceivable that such biological effects could potentially reduce micromotion at the fracture site even before macroscopic structural stability is fully realized, thereby possibly contributing to symptomatic relief despite the radiographic progression of the fracture.

The apparent discrepancy between our findings and previous studies can be explained through a temporal analysis of fracture healing. While these earlier reports highlighted the biological potential of anabolic agents in enhancing stability, our results reflect a critical temporal mismatch between pharmacological onset and the rapid mechanical failure observed in the acute phase [13,19]. In our cohort, despite the prompt initiation of medication upon diagnosis, the most significant progression of collapse occurred within the first two weeks. This suggests that the rate of mechanical collapse outpaces the time required for pharmacological agents to achieve sufficient bone mineralization and structural stiffness. Ultimately, this underscores that during the acute ‘mechanical window’ of instability, initial fracture characteristics are more decisive factors for height loss than the biological stimulus provided by early medication.

Although the medications did not differ significantly in preventing structural collapse, significant differences were observed in clinical outcomes regarding pain relief. Specifically, the RM group demonstrated a distinct advantage in pain management, maintaining significantly lower pain scores throughout the follow-up period compared to the control group in the LMM analysis. While the precise mechanism remains unclear within the scope of this study, this finding might be related to the unique dual action of RM, which simultaneously promotes bone formation and inhibits resorption [20]. Previous literature suggests that such anabolic effects could potentially contribute to the earlier stabilization of micro-fractures within the vertebral body [23]. In this context, our observation implies that RM could be a favorable option for improving the patient’s quality of life through effective pain relief in the acute phase, even if structural progression is not fully prevented. This biological potential is further supported by recent preclinical evidence demonstrating that romosozumab significantly enhances spinal implant stability and osseointegration in osteoporotic bone models [24].

Our findings strongly support the prognostic value of the Sugita classification, first proposed to predict the risk of delayed union and collapse [10]. Consistent with Sugita et al.’s original observations, we identified the ‘Unstable type’ (swelled, projecting, and dented) as a major risk factor for rapid vertebral collapse. While previous studies have demonstrated that TPTD can accelerate fracture healing and promote union in osteoporotic vertebral fractures [13], our data indicates that the mechanical instability of these fracture types often outweighs the biological effects of medication in the acute phase. The precipitous collapse observed in the Unstable group within the first two weeks underscores that pharmacological treatment alone is insufficient for these high-risk morphotypes. Therefore, for patients presenting with ‘Unstable’ morphology, clinicians should consider more aggressive closed management strategies, including stricter immobilization and closer radiographic monitoring, to promptly identify progressive collapse and prevent severe deformity, regardless of the prescribed osteoporosis medication.

This study has several limitations that should be considered when interpreting our findings. First, the retrospective design and the requirement for a minimum one-year follow-up likely introduced selection bias. Specifically, this inclusion criterion may have introduced a survivorship bias, as it potentially excluded the most severe or complicated cases—such as patients who might have undergone early surgical intervention, experienced mortality, or been lost to follow-up due to poor clinical status. While we cannot account for these missing data points due to the retrospective nature of this study, this exclusion could have attenuated the observed differences between treatment groups. Additionally, the significantly higher initial VCR in the DMAB group reflects real-world clinical practices where socioeconomic factors, such as insurance coverage and patient preference, heavily influenced treatment allocation rather than randomization. While we utilized adjusted linear mixed models (LMMs) to control these baseline imbalances, the possibility of residual confounding remains.

Second, although our intra-observer reliability was excellent (ICC: 0.96), all radiographic assessments were performed by a single observer. Furthermore, the exclusive use of supine radiographs represents a significant limitation of this study. This imaging protocol was initially prioritized to ensure patient safety and prevent potential fracture aggravation during the acute phase, where severe pain often precluded weight-bearing standing views. To maintain methodological consistency and ensure uniformity across the serial 3-month follow-up, supine measurements were utilized throughout the study period. However, we fully acknowledge that this static assessment may fail to capture dynamic instability, potentially resulting in an underestimation of the true extent of vertebral collapse compared to weight-bearing radiographs.

Third, the relatively small sample sizes for specific fracture morphology subtypes—such as the Swelled (*n* = 9) and Dented (*n* = 8) types—necessitated their dichotomization into broader ‘unstable’ and ‘compressive’ categories. While this categorization was biomechanically justified based on prognostic implications for instability, it inherently reduced the statistical power to detect more nuanced, subtype-specific interactions between pharmacological efficacy and individual fracture morphologies.

Fourth, the 3-month observation window for vertebral height loss and 1-year follow-up for BMD may be relatively short. This highlights a fundamental distinction between the acute biomechanical collapse phase—which occurs rapidly over days and weeks—and the biological bone remodeling phase, which requires several months to years to achieve structural rigidity. Therefore, our null structural results should be interpreted as a limited effect on acute mechanical failure rather than an indicator of long-term pharmacological inefficacy for osteoporosis treatment.

Fifth, medication adherence was verified through prescription records rather than objective measures such as injection logs. While this reflects the pragmatic reality of a retrospective study, potential variations in individual compliance within the initial window remain an inherent limitation. Additionally, the absence of functional outcome metrics, such as the Oswestry Disability Index (ODI), limits our ability to correlate radiographic changes with clinical disability.

Lastly, this was a single-center study with a limited sample size, and our results demonstrate associations rather than definitive causal links regarding medication efficacy. The identification of IVC formation was also based on an operational definition due to the short timeframe. Future large-scale prospective studies are warranted to validate these findings.

## 5. Conclusions

In conclusion, our findings indicate that short-term osteoporosis medication is not significantly associated with a reduction in vertebral height loss during the initial 3-month acute phase of OVCFs. However, romosozumab demonstrated a favorable association with clinical pain relief, potentially improving patient quality of life during the early recovery period despite ongoing structural changes. Ultimately, the progression of vertebral collapse was principally determined by baseline structural characteristics—a specifically unstable fracture morphology and the ‘floor effect’ related to initial compression severity—rather than the pharmacological regimen. Therefore, clinical management should prioritize early imaging-based risk stratification to identify patients at high risk for progressive deformity. For those presenting with unstable morphotypes or low initial compression, proactive stabilization strategies should be prioritized, acknowledging the limited role of immediate pharmacological intervention in preventing acute mechanical failure.

## Data Availability

The data presented in this study are available on request from the corresponding author. The data are not publicly available due to ethical and privacy restrictions, as they contain sensitive patient information protected under the Institutional Review Board (IRB) of Daejeon Sun Hospital.

## Abbreviations

OVCF	Osteoporotic vertebral compression fracture
VCR	Vertebral compression rate
LMM	Linear mixed model
BMD	Bone mineral density
VAS	Visual analogue scale
DMAB	Denosumab
TPTD	Teriparatide
RM	Romosozumab
MRI	Magnetic resonance imaging
CT	Computed tomography
TLSO	Thoracolumbar orthosis
IVC	Intravertebral vacuum cleft
DEXA	Dual-energy X-ray absorptiometry
